# Negative prognostic impact of tumor deposits in stage III colorectal cancer patients

**DOI:** 10.1371/journal.pone.0310327

**Published:** 2024-09-26

**Authors:** Ting Ma, Zhaofu Qin, Guohui Xu, Peng-Wen Zheng, Longhai Feng, Dening Ma, Zhixuan Fu, Xinyi Gao

**Affiliations:** 1 Postgraduate Training Base Alliance of Wenzhou Medical University (Zhejiang Cancer Hospital), Hangzhou, Zhejiang Province, China; 2 Department of Colorectal Surgery, Zhejiang Cancer Hospital, Hangzhou, Zhejiang Province, China; 3 Department of Radiology, Zhejiang Cancer Hospital, Hangzhou, Zhejiang Province, China; West China Hospital of Sichuan University, CHINA

## Abstract

**Background:**

The prognostic value of tumor deposits (TDs) in stage III colorectal cancer (CRC) patients is poorly described based on the current tumor node metastasis (TNM) stage system.

**Materials and methods:**

Based on the data from the Surveillance, Epidemiology, and End Result (SEER) database between 2010 to 2020 and local hospital between 2006 to 2022, the clinicopathological features of stage III CRC patients with TDs were screened by Chi-square test. Kaplan-Meier curves were performed to describe the significant difference in overall survival (OS) among the different groups, and log-rank tests were used to compare the cumulative survival distributions.

**Result:**

Patients with TDs exhibited more aggressive tumors, characterized by advanced T staging (T3&T4), N staging (N2), perineural invasion, and more advanced TNM stage. The presence of TDs was identified as a negative prognostic factor in stage III CRC patients, with the co-existence of TDs and lymph node metastasis associated the poorest prognosis. A pairwise comparison revealed no statistically significant difference between TD+N1a/b and N1c groups, while the OS of TD-LN+ (TD- N1a/b) patients was the most favorable within the N1 stage. Notably, patients with a single lymph node positive had a significantly better OS than those with a single TD positive.

**Conclusion:**

The presence of tumor deposits was a negative prognostic factor in stage III colorectal cancer patients, and the significance of tumor deposits was underestimated in the current TNM staging system.

## 1.Introduction

Colorectal cancer (CRC) is the third most commonly diagnosed cancer and the second most frequent cause of cancer‐related death worldwide [1–3]. The occurrence and mortality of CRC has declined in some European and northern American countries, but the incidence and mortality of CRC continues to rise in China [3,4]. In addition to age, recognized risk factors for colorectal cancer include genetics (such as family history of colorectal cancer), lifestyle (such as smoking, alcohol consumption, diet, and lack of physical activity), gender, race, and ethnicity [5]. For example, diet influences the development of colorectal cancer through direct effects on immune response and inflammation, as well as indirect effects on risk factors for overnutrition and obesity in colorectal cancer, and the gut microbiome also plays an important role in the relationship between diet and cancer [6]. In addition, a large number of studies have shown that patients with diabetes have a significantly increased risk of developing colorectal cancer, suggesting that diabetes prevention and control may reduce the occurrence of CRC [7,8] Inflammation affects all stages of tumorigenesis, and a key signaling pathway for inflammation is through activation of the caspase-1 inflammasome. It has been shown that activation of inflammasome and IL-18 signaling pathway has a strong protective effect in colitis-associated colorectal cancer [9], among which NOD-like receptor protein 3 (NLRP3) is one of the most studied inflammasome. STING signaling is known to promote the production of interleukin-18 and IL-1β by macrophages through activation of NLRP3, mediating the anti-tumor function of NK cells to eliminate colorectal cancer liver metastases [10]. To guide treatment strategies and predict prognosis, the American Joint Committee on Cancer (AJCC) tumor node metastasis (TNM) staging system is developed and constantly updated. Tumor deposits (TDs), defined as discrete tumor nodules without lymph structure within the area of regional lymph node in primary tumor [11–13], have been added into nodal staging. In 8th edition staging system, stage III patients with TDs are classified as N1c only if they have no lymph node (LN) metastases.

Actually, several studies indicated that the prognostic value of TDs has been underestimated [14–16]. When lymph node metastases (LNMs) coexist with TD, the N staging for N1a/b and N2a/b patients only consider the status and account of positive LN. Ignoring the status of TDs for N1a/b and N2a/b patients might underestimate the risk of stage III patients and result in inadequate adjuvant treatment [14]. Recent studies have shown that TDs should be defined as metastatic lymph nodes (LNs) [17–19]. Increasingly scholars supported that TDs was a signal of distant metastasis and the status and quantity of TDs were strongly related to the overall survival of patients with CRC [20,21]. Therefore, more further studies are urgently needed to investigate the value of TDs in the staging of colorectal cancer.

In this study, to determine the prognostic values of TDs in stage III colorectal cancer, we conduct a comprehensive statistical analysis based on the data from the Surveillance, Epidemiology and End Results (SEER) and local hospital.

## 2. Materials and methods

### 2.1 Data source and study subjects

**Fig 1:**
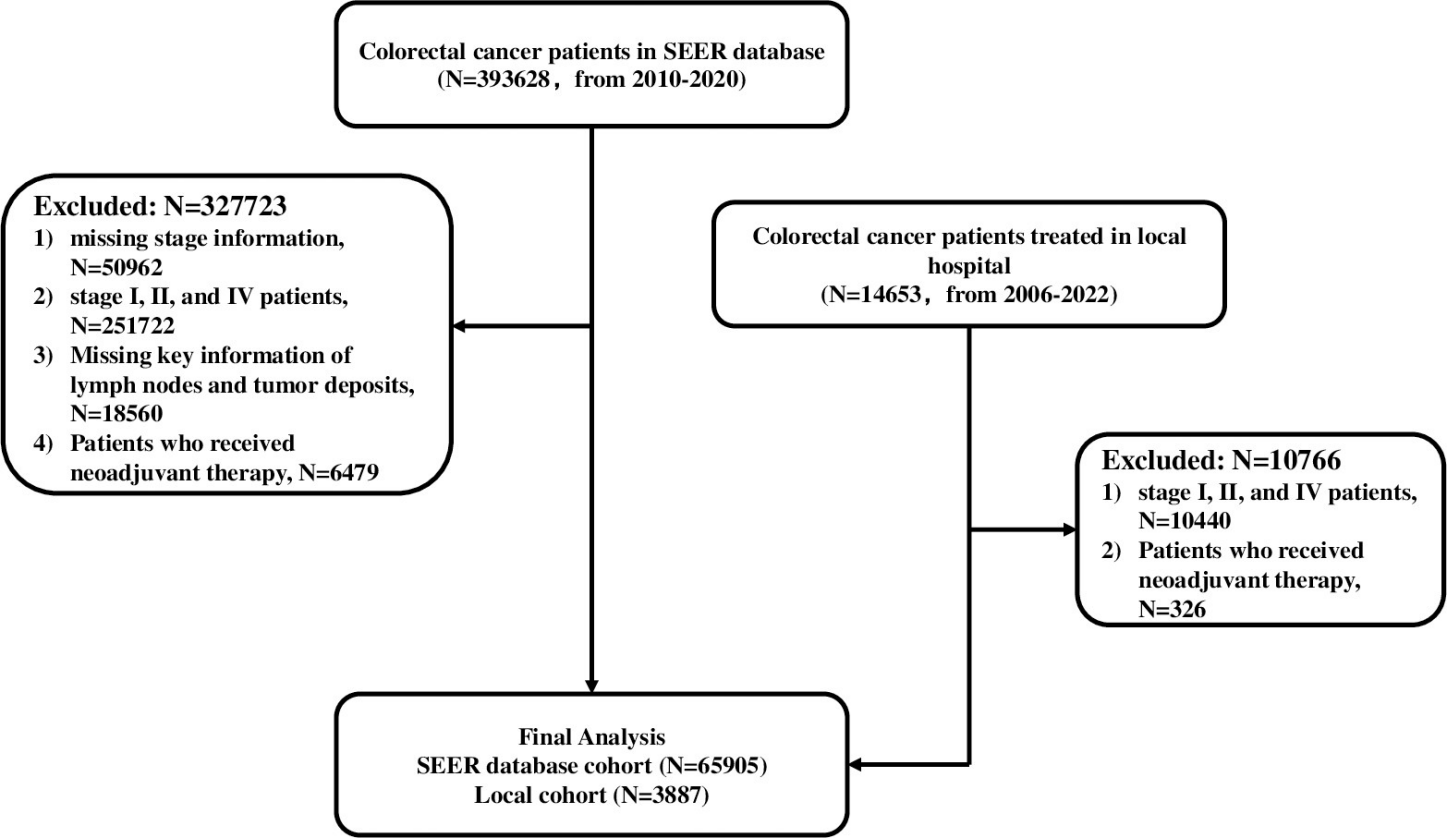
Flowchart of participant inclusion and exclusion.

The SEER program, established by the United States National Cancer Institute (NCI), aims to comprehensively collect clinicopathological data on various cancer types, is used to analyze specific cancer associated incidence, prevalence, and prognosis [22]. Patients included in this study must be diagnosed with CRC from 2010 to 2020 in SEER database. Patients with uncomplete TNM stage and stage I, II, III were excluded. Those who missed key information of lymph nodes and tumor deposits were excluded. Patients who received neoadjuvant therapy were excluded to control the result deviation caused by the descending stage of the primary lesion after neoadjuvant therapy. Similarly, among the CRC patients with stage III treated in local hospital from 2006 to 2022, those who received neoadjuvant therapy were excluded, and the rest consisted of the local cohort. The present research was approved by the research ethics committee of Zhejiang Cancer Hospital (IRB-2023-562). We had access to information that could identify individual participants during or after data collection. A detailed flowchart diagram is shown in Fig 1. All patients in our study were staged according to the AJCC staging principles. In this study, we used 5-year overall survival (OS) as the primary endpoint. OS is the time between the date of diagnosis and the date of death from any cause or the last follow-up visit. Due to SEER database is public-available, no need for this study to acquire the informed consent or institutional review board approval. And the data of local cohort in this study has acquired the approval of local hospital. Both in SEER cohort and local cohort, patients with stage III were divided into 3 groups based on the results of pathology: LN+TD–, LN+TD+, and LN–TD+.

### 2.2 Statistical analysis

R (version 4.2.1) was used for statistical analysis, and P-value < 0.05 was defined as statistically significant. Chi-square test was used to detect the clinicopathological features among 3 groups. Next, the clinicopathological characteristics significantly related to survival were screened by univariate Cox regression analysis. Subsequently, the variables which significantly related to survival were further screened by multivariate Cox regression analysis. Univariate and multivariable Cox regression analysis results were summarized using hazard ratios (HRs) and 95% confidence intervals (CIs). Furthermore, Kaplan-Meier curves were performed to describe if there were the significant difference in overall survival among the different groups, and log-rank tests were used to compare the cumulative survival distributions.

## 3.Result

### 3.1 Patient characteristics

A total of 393,628 patients were diagnosed with CRC from 2010 to 2020 in the SEER database. However, for the purpose of this analysis, only 65,905 patients from the SEER cohort and 3,887 patients from the local database were included. The baseline demographic and clinicopathological characteristics of the patients in the SEER database are listed in Table 1, while the information for the local database can be found in S1 Table. In the SEER cohort, patients were categorized into three groups: TD-LN+ (48680, 73.86%), TD+LN- (3,653, 5.54%), and TD+LN+ (13572, 20.59%). As shown in Table 1, patients in the TD+LN+ group had worse tumor biological behavior, characterized by an increased prevalence of T3&T4, N2, stage IIIC, and perineural invasion. Furthermore, this group of patients demonstrated the highest proportion of preoperative CEA positivity in the three groups, and were more likely to undergo adjuvant chemotherapy and extensive LN dissection (≥12). While in the local database, patients were divided into three groups: TD-LN+ (2,627, 67.58%), TD+LN- (285, 7.33%), and TD+LN+ (975, 25.08%). The findings from the local cohort align with those observed in the SEER cohort, as presented in S1 Table. In summary, the aforementioned findings indicated a strong association between TD+LN+ patients and unfavorable clinicopathological characteristics.

**Table 1:** The comparison of clinicodemographic data for CRC patients with stage III in SEER cohort.

Characteristics	Overall N = 65905(%)	Tumor deposits and Lymph nodes metastasis			*P*-value
		TD-LN+ N = 48680(%)	TD+LN- N = 3653(%)	TD+LN+ N = 13572(%)	
**Gender**					0.159
Female	32748(49.7)	24290(49.9)	1812(49.6)	6646(49.0)	
male	33157(50.3)	24390(50.1)	1841(50.4)	6929(51.0)	
**Age (years)**					<0.001
<65	28483(43.2)	21026(43.2)	1449(39.7)	6008(44.3)	
≥65	37422(56.8)	27654(56.8)	2204(60.3)	7564(55.7)	
**Race**					<0.001
Yellow	6521(9.9)	4797(9.9)	392(10.7)	1332(9.8)	
White	50956(77.3)	37531(77.1)	2803(76.7)	10622(78.3)	
Black	7502(11.4)	5711(11.7)	399(10.9)	1392(10.3)	
Unknown	926(1.4)	641(1.3)	59(1.6)	226(1.7)	
**Site**					<0.001
Left colon	27122(41.2)	19478(40.0)	1729(47.3)	5915(43.6))	
Right colon	33886(51.4)	25767(52.9)	1572(43.0)	6547(48.2)	
Rectum	4897(7.4)	3435(7.1)	352(9.6)	1110(8.2)	
**T stage**					<0.001
T1&T2	9383(14.2)	8340(17.1)	349(9.6)	694(5.1)	
T3&T4	56505(85.7)	40326(82.8)	3303(90.4)	12876(94.9)	
Unknown	17(0)	14(0.1)	1(0)	2(0)	
**N stage**					<0.001
N1	44387(67.3)	34250(70.4)	3650((99.9)	6487(47.8)	
N2	21504(32.6)	14417(29.6)	2(0.1)	7085((52.2)	
Unknown	14(0)	13(0)	1(0)	0(0)	
**TNM stage**					<0.001
IIIA	8215(12.5)	7398(15.2)	350(9.6)	467(3.4)	
IIIB	42543(64.5)	31931(65.6)	2942(80.5)	7670(56.5)	
IIIC	15147(23.0)	9351(19.2)	361(9.9)	5435(40.0)	
**Perineural invasion**					<0.001
Positive	12288(18.6)	7033(14.4)	699(19.1)	4556(33.6)	
Negative	50101(76.0)	38976(80.1)	2770(75.8)	8355(61.6)	
Unknown	3516(5.4)	2671(5.5)	184(5.0)	661(4.9)	
**Preoperative CEA**					<0.001
Positive	17969(27.3)	12693(26.1)	1035(28.3)	4241(31.2)	
Negative	24063(36.5)	18397(37.8)	1254(34.3)	4412(32.5)	
Unknown	23873(36.2)	17590(36.1)	1364(37.3)	4919(36.2)	
**Adjuvant chemoradiotherapy**					<0.001
Yes	42133(63.9)	31291(64.3)	1978(54.1)	8864(65.3)	
No	23772(36.1)	17389(35.7)	1675(45.9)	4708(34.7)	
**LN Dissection**					<0.001
≤11	6212(9.4)	4287(8.8)	535(14.6)	1390(10.2)	
≥12	55986(84.9)	41692(85.6)	2856(78.2)	11438(84.3)	
Unknown	3707(5.7)	2701(5.6)	262(7.2)	744(5.5)	

### 3.2 Characteristics related to survival outcomes of stage III CRC patients

As total of nine variables were found to have a significant relationship with survival in the SEER cohort through univariate analysis (P<0.05) (Table 2). These variables consisted of gender, age, T stages, N stages, TDs, perineural invasion, preoperative CEA, adjuvant chemotherapy, and LN dissection. Subsequently, all variables included in the multivariate COX analysis exhibited statistical significance (P<0.001). In terms of overall survival (OS), males exhibited a slight disadvantage compared to females (HR: 1.150, 95%CI: 1.112–1.190). Additionally, patients aged 65 years and older experienced poorer survival compared to younger individuals (age < 65 years) (HR: 2.013, 95% CI: 1.936–2.092). Patients with advanced T stages (T3 and T4) had a poorer survival rate (HR: 1.662, 95%CI: 1.561–1.770). Besides, CRC patients with N1 had a better survival rate than patients with N2 (HR:1.656, 95%CI: 1.599–1.716). Patients with TDs showed a worse overall survival (HR: 1.371, 95%CI: 1.321–1.424). Additionally, patients with positive CEA (HR: 1.339, 95%CI: 1.285–1.395) or perineural invasion (HR: 1.406, 95%CI: 1.359–1.455) had worse survival than those with negative CEA or no perineural invasion. It was worth noting that patients who received adjuvant chemotherapy had a better survival rate than those who did not (HR: 0.375, 95%CI: 0.362–0.388). Furthermore, a higher lymph node dissection (≥12) was associated with a better survival outcome compared to a lower number of dissections (≤11) (HR: 0.637, 95%CI: 0.605–0.671). Similarly, in the univariate COX analysis of the local cohort, as shown in S2 Table, the results supported these findings. In the univariate COX analysis, all variables other than gender (P = 0.654), schistosomiasis (P = 0.641), hepatitis (P = 0.445), diabetes (P = 0.340), hypertension (P = 0.129) and LN dissection (P = 0.083) were related to overall survival and were included in multivariate COX analysis. Also, multivariate COX analysis identified the seven variables included were related to survival (P<0.05). Collectively, these results suggested that several characteristics were relative to poor survival outcomes in CRC patients.

**Table 2:** Overall univariate and multivariate analysis for CRC patients with stage III in SEER cohort.

Characteristics	Univariate COX regression			Multivariate COX regression		
	HR	CI	*P* value	HR	CI	*P* value
**Gender**						
Female	1			1		
male	1.032	1.007–6.417	0.011	1.150	1.112–1.190	<0.001
**Age (years)**						
<65	1			1		
≥65	2.576	2.506–2.647	<0.001	2.013	1.936–2.092	<0.001
**T stage**						
T1&T2	1			1		
T3&T4	2.148	2.056–2.243	<0.001	1.662	1.561–1.770	<0.001
**N stage**						
N1	1			1		
N2	1.557	1.538–1.616	<0.001	1.656	1.599–1.716	<0.001
**Tumor Deposits**						
No	1			1		
Yes	1.544	1.504–1.586	<0.001	1.371	1.321–1.424	<0.001
**Perineural invasion**						
Negative	1			1		
Positive	1.515	1.470–1.561	<0.001	1.339	1.285–1.395	<0.001
**Preoperative CEA**						
Negative	1			1		
Positive	1.655	1.603–1.708	<0.001	1.406	1.359–1.455	<0.001
**Adjuvant chemoradiotherapy**						
No	1			1		
Yes	0.345	0.336–0.353	<0.001	0.375	0.362–0.388	<0.001
**LN Dissection**						
≤11	1			1		
≥12	0.662	0.638–0.687	<0.001	0.637	0.605–0.671	<0.001

### 3.3 The effect of TDs and LNs stratified by chemotherapy in stage III CRC patients

**Fig 2:**
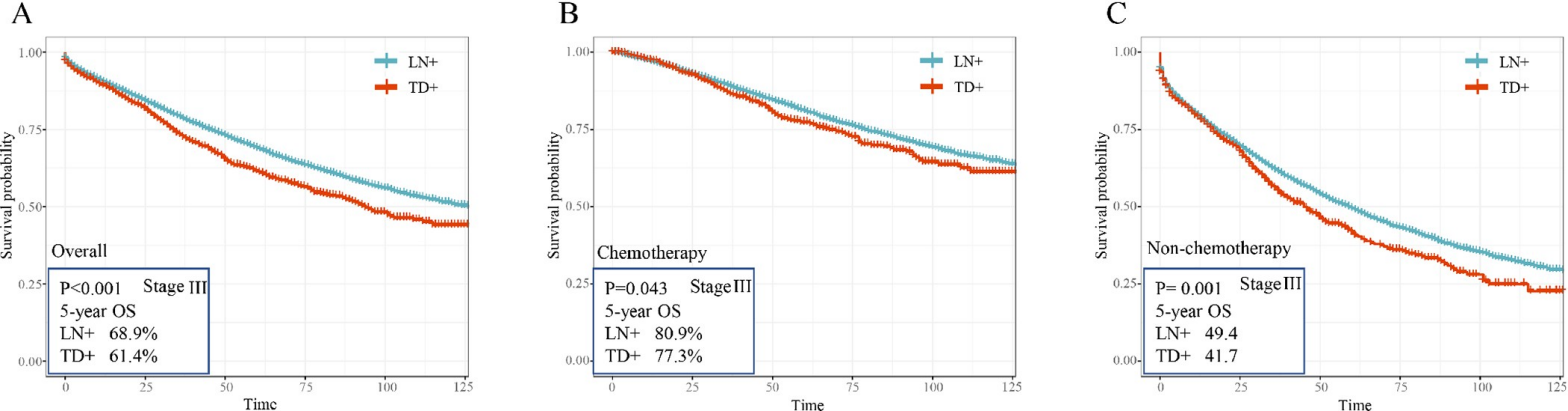
Overall survival by single TD+ and single LN+ in stage III stratified by chemotherapy. The Kaplan-Meier analysis was performed to access the overall survival difference between single LN positive patients and single TD positive patients (A). Next, the Kaplan-Meier analysis was conducted to determine the impact of single TD and single LN stratified by chemotherapy (B, C).

Additionally, the S1 Fig demonstrated that all stage III patients (including stage IIIA, IIIB and IIIC) who received chemotherapy experienced an overall survival benefit (P<0.001). Subsequently, in order to assess the impact of tumor deposits and lymph nodes stratified by chemotherapy, the KM analysis was performed to examine the disparity in overall survival between CRC patients with single positive lymph node and those with single positive tumor deposit. The outcomes were presented in Fig 2. Fig 2A showed that patients with single positive lymph node had a significantly better overall survival than patients with single positive tumor deposit (5-years OS: 68.9% vs. 61.4%, P<0.001). Furthermore, Fig 2B and 2C revealed that patients with single positive tumor deposit had a lower 5-year overall survival rate in comparison to patients with single positive lymph node, regardless of whether they received adjuvant chemotherapy. Altogether, compared to the status of LN+, TD+ correlated with significantly worse survival, and the prognostic value of TDs was underestimated.

### 3.4 The effect of TDs and LNs in stage III CRC patients

**Fig 3:**
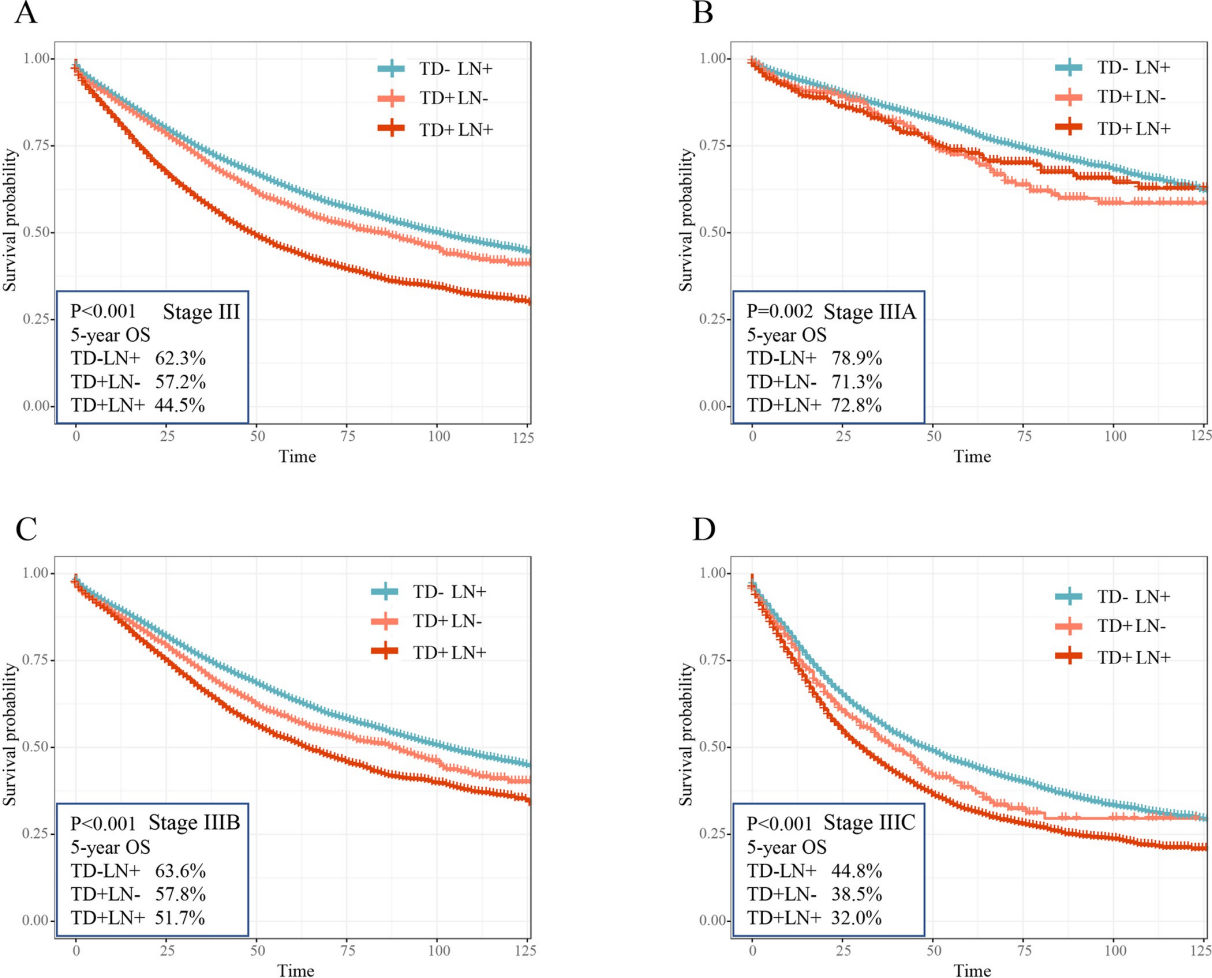
Overall survival by the status of TDs and LNs in stage III subgroups. Among the CRC patients with stage III (A), IIIA (B), IIIB (C) and IIIC (D) in SEER cohort, the Kaplan-Meier curves were performed to describe the significant difference in overall survival among the different groups including TD+LN+, TD+LN- and TD-LN+, and log-rank tests were used to compare the cumulative survival distributions.

**Fig 4:**
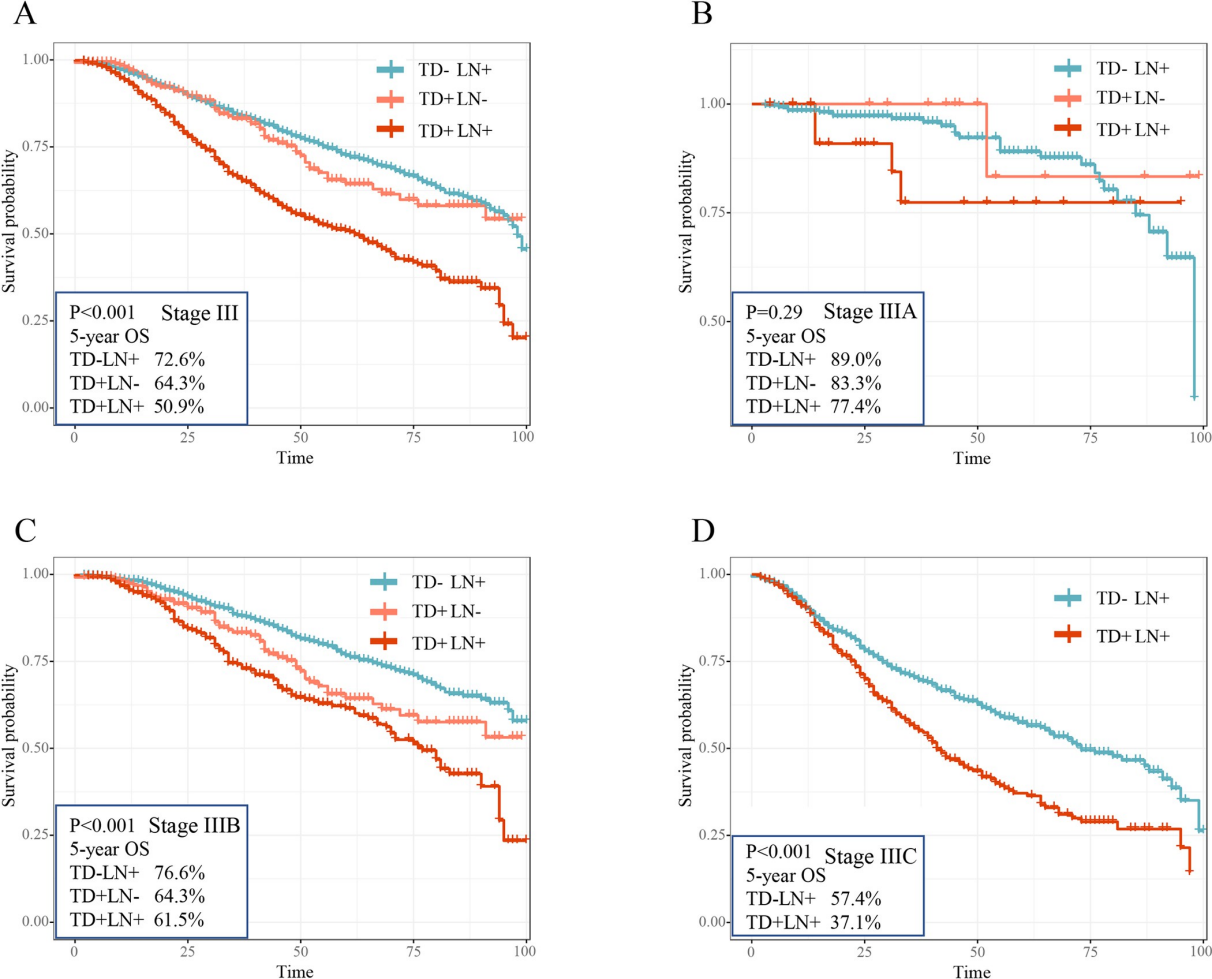
Overall survival by the status of TDs and LNs in stage III subgroups. Among the CRC patients with stage III (A), IIIA (B), IIIB (C) and IIIC (D) in local cohort, the Kaplan-Meier curves were performed to describe the significant difference in overall survival among the different groups including TD+LN+, TD+LN- and TD-LN+, and log-rank tests were used to compare the cumulative survival distributions.

According to the conventional TNM staging system, the N stage can be divided into five subgroups (N1a, N1b, N1c, N2a, and N2b) based on the number of positive lymph nodes and TD status. However, the significance of TD status and its quantity on the prognosis of CRC patients has been underestimated. To evaluate the impact of TDs status in stage III CRC patients, a Kaplan-Meier analysis was conducted, stratifying the patients based on the different TNM stage III classifications. The results of SEER cohort and local cohort were shown in the Figs 3 and 4, respectively. As depicted in Fig 3A, 3C and 3D, among overall stage III, stage IIIB and IIIC patients in SEER cohort, patients in the TD+LN+ group had the poorest 5-years overall survival rates (overall stage III: 44.5%; stage IIIB: 51.7%; stage IIIC: 32.0%), followed by TD+ LN- group (overall stage III: 57.2%; stage IIIB: 57.8%; stage IIIC: 38.5%), while TD-LN+ group demonstrated the most favorable outcomes (overall stage III: 62.3%; stage IIIB: 63.6%; stage IIIC: 44.8%). Interestingly, Fig 3B showed that stage IIIA patients with TD+ LN- had the worst overall survival, TD+ LN+ patients were in the middle, and TD- LN+ patients had the best overall survival, the estimated 5‐year OS rates were 71.3%, 72.8% and 78.9% for the respective groups. Notably, the validation of these findings was conducted in the local cohort, as illustrated in Fig 4, through Kaplan-Meier analysis. Similar findings were observed in both overall stage III (TD+ LN+: 50.9%; TD+ LN-: 64.3%; TD- LN+: 72.6%) and stage IIIB (TD+ LN+: 61.5%; TD+ LN-: 64.3%; TD- LN+: 76.6%) patients. However, due to insufficient data, the aforementioned conclusions could not be confirmed for stage IIIA and IIIC diseases. Compared with other groups, these results indicated that all stage III CRC patients with TD+LN+ apart from stage IIIA had the poorest overall survival.

### 3.5 The effect of TDs and LNs in stage IIIA CRC patients

**Fig 5:**
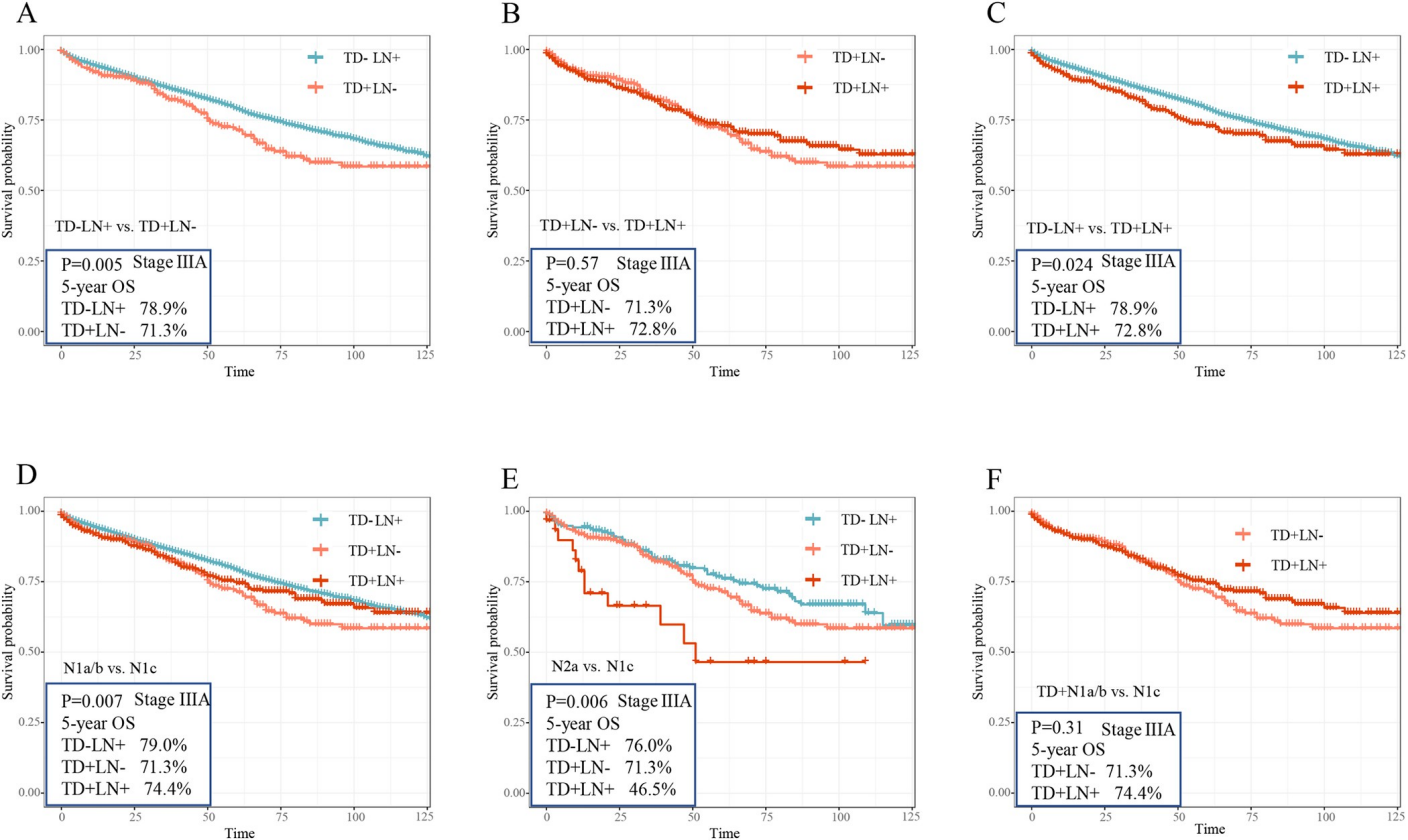
Overall survival by the status of TDs and LNs in stage IIIA subgroups. According to TNM staging, the N stages of IIIA include N1a, N1b, N1c and N2a. In SEER cohort, a pairwise comparison of the three groups (A, B, C), including TD+LN+, TD+LN- and TD-LN+, was conducted in overall stage IIIA was conducted to describe the significant difference in overall survival. Also, stage IIIA LN positive patients were divided into N1a/b (D, F) and N2a (E) groups and the Kaplan-Meier analysis was conducted to access the impact of TDs in two groups.

To further determine the impact of TDs status, the Kaplan-Meier analysis was performed to assess the influence of TDs and LNs in CRC patients with TNM stage IIIA in the SEER cohort. According to TNM staging, the N stages of IIIA include N1a, N1b, N1c and N2a. As depicted in Fig 5A–5C, a pairwise comparative analysis was performed on the three groups in the stage IIIA, revealing that group TD-LN+ exhibited the best overall survival (5-years OS: 78.9%) rate. Notably, there was no statistically significant difference between group TD+LN+ and group TD+LN- (5-years OS: 71.3% vs. 72.8%, P = 0.57). Next, stage IIIA patients with positive lymph nodes were further categorized into N1a/b and N2a groups, and the impact of TDs in these two groups was assessed using the Kaplan-Meier analysis. Fig 5D demonstrated that patients of TD+LN- (N1c) group had the worst survival outcomes, followed by patients of TD+LN+ (TD+ N1a/b) group, while patients of TD-LN+ (TD- N1a/b) group exhibited the best overall survival in the N1 stage (5-years OS: 71.3% vs. 74.4% vs. 79.0%, P = 0.007). Meanwhile, the KM curves showed in Fig 5E indicated that patients of TD+N2a group had the poorest overall survival, while TD+LN- patients fell in the intermediate range, and TD-N2a patients had best survival (5-years OS: 46.5% vs. 71.3% vs. 76.0%, P = 0.006). However, the pairwise comparison analysis revealed no statistically significant difference between the TD+N1a/b and TD+N- (N1c) groups (5-years OS: 71.3% vs.74.4%, P = 0.31), as illustrated in Fig 5F. Taken together, these results demonstrated that among stage IIIA patients, only N1a/b stage patients with TDs compared to patients with N1c stage had no difference in 5-years overall survival.

## 4.Disscusion

In our study, we found that the guiding values of TDs were significantly underestimated in the treatment strategies of CRC patients with stage III, as shown in previous studies [13,23–25]. Only the tissue without lymph structure, which located in the peri-colorectal adipose tissue lymphatic drainage territory of primary tumor, were defined as TDs [11,13]. First of all, this study showed that 26.13% of stage III patients had TDs in SEER cohort and 32.41% of CRC patients with stage III had TDs in local cohort. Therefore, ignoring the role of TDs in LN-positive patients with stage III might be terrible, which were likely to result in inadequate adjuvant therapy. Both in SEER cohort and local cohort, patients with TD+LN+ had more malignant tumors, including advanced T staging (T3&T4), N staging (N2) and TNM stage (stage IIIC) [24]. Additionally, compared to other two groups, TD+LN+ patients exhibited a highest proportion of preoperative CEA positive [24]. Furthermore, TDs were more likely to occur in the patients with perineural invasion, which aligns with findings from previous studies [14,26,27]. Above all, patients with TD+LN+ were more likely to suffer from more aggressive tumor, which might explain those patients were more likely to received adjuvant chemoradiotherapy and more LN dissection (≥12).

In SEER cohort, 9 variables, including gender, age, T stages, N stages, TDs, perineural invasion, preoperative CEA, adjuvant chemoradiotherapy and LN dissection, were significantly related to survival through univariate analysis. Furthermore, the multivariate COX analysis exhibited that male, old (age ≥ 65 years), advanced T staging (including T3 and T4) and N staging (N2), positive CEA and perineural invasion were related to worse overall survival. It was worth noting that patients treated with adjuvant chemoradiotherapy and more LN dissection (≥12) resulted a better survival outcome. Also, the similar conclusions were acquired in local cohort.

Actually, our study indicated that the presence of TD appeared to be as important as the prognostic values of N status [28] Among overall stage III, stage IIIB and IIIC patients in SEER cohort, patients with TD+LN+ had the worst 5-years overall survival, followed by TD+ LN-, and TD-LN+ had the best 5-year OS. Interestingly, stage IIIA patients with TD+ LN- had the worst overall survival, TD+ LN+ patients were in the middle, and TD- LN+ patients had the best overall survival. Notably, in local cohort, the similar results were obtained in overall stage III and stage IIIB patients, but for stage IIIA and IIIC diseases could not validate the above conclusions due to insufficient data. Above conclusions indicated that the presence of TDs was a poor prognostic factor in stage III patients with CRC, as evidenced by previous studies [14,27,29,30]. TD patients have a significantly higher risk of death regardless of LNM status [31] and the concomitant presence of TDs and LNM carried poor prognosis [32]. The origin of TDs remained uncertain and might be various [14]. TDs were combined with perineural, perivascular and intravascular origin [33,34]. Previous studies have shown that the optimal threshold for TD counts in the SEER database is 4 [11], with a significant decrease in 5-year OS for TD≥4, and that the optimal threshold for TD counts in the TD-positive subgroup during the N2 period is 5 [35], with a significant decrease in both OS and CSS for 5-year TD≥5. However, in our study, patients with single positive TD had a lower 5-year overall survival rate in comparison to patients with single positive LN. The worse overall survival of patients with TDs compared to patients with LN positive alone might be attributed to the presence of vessels and nerves in the majority of TDs [14].

To determine the reason for the difference in outcomes between patients with IIIA and the overall stage III population, the Kaplan-Meier analysis was performed to further analyze the stage IIIA subgroups. According to TNM staging, the N stages of IIIA include N1a, N1b, N1c and N2a. A pairwise comparison of the three groups among overall stage IIIA patients indicated that patients with TD-LN+ had the best overall survival, with no statistical difference between patients with TD+LN+ and TD+LN-. Stage IIIA patients with LN positive could be divided into N1a/b and N2a groups. Patients with TD+N2a had the worst overall survival, followed by TD+LN- patients, and TD-N2a patients had best OS. Interestingly, the pairwise comparison found no statistical difference between TD+N1a/b and N1c groups, and the overall survival of TD-LN+ (TD- N1a/b) patients was best in the N1 stage. Above conclusions were consistent with previous study [23]. Patients with TDs were related to more lympho-vascular and perineural invasion, and TDs have been considered as the independent predictors of liver, lung, and peritoneal metastases [1].

To more intuitively compare the prognostic values of TD and LN in CRC patients with stage III, we conducted Kaplan-Meier analysis to access the overall survival difference between single LN positive patients and single TD positive patients. Patients with single LN positive had a significantly better overall survival than patients with single TD positive, regardless of receiving adjuvant chemotherapy. The behavior of tumor cells in TDs was similar to that of tumor buds, which migrated over and through histological boundaries [24,36]. Tumor cells in TDs re-acquired the mesenchymal-like characteristics indicated a significant phenotypic plasticity. This might explain the worse overall survival of patients with TDs, since the ability to rapidly and repeatedly undergo the process of both EMT and MET is necessary for the growth and spread of tumor cells [36–38]. Also, the latest clinical practice guidelines developed by the American Society of Colon and Rectal Surgeons has advocated N1c stage patients should receive adjuvant chemotherapy [23]. Previous studies have demonstrated a survival benefit for N1c patients to treat adjuvant therapy [1].

The inflammasome has a protective effect on colitis-associated colorectal cancer, and in particular NLRP3 is closely associated with colorectal cancer [39]. Previous studies have shown that NLRP3 deficiency is associated with a significantly increased incidence of colitis and colorectal cancer induced by the DNA-damaging agent azomethane (AOM) and the chemical colitogenic dextran sulfate sodium (DSS) in mouse models [40,41]. Therefore, increasing or decreasing the activity of inflammasome and its effector molecules may have a potential therapeutic effect on colorectal cancer. In addition, numerous studies have shown that diets recommended by the World Cancer Research Fund (WCRF) and the American Institute for Cancer Research (AICR) reduce the risk of high cholesterol, high blood sugar, and obesity through various pathways mediated by the NLRP3 inflammasome and adenosine 5’-adenosine monophosphate (AMP) activated protein kinase (AMPK), also enhance cardiorenal fitness and decrease systemic inflammation [39]. Our study has demonstrated that TD is an independent risk factor for colorectal cancer. While adopting an anti-inflammatory dietary pattern may be beneficial for high-risk populations in preventing CRC, it remains unclear whether such a diet will positively affect TD status and prognosis in CRC patients. However, we believe that in future prospective studies, more and more scholars will further explore the relationship between anti-inflammatory diet and colorectal cancer occurrence, TD status and prognosis.

Here were several major limitations in our study. Firstly, the source of TNM staging in the SEER database is unclear, which can lead to potential bias. Secondly, we did not consider the prevalence of neoadjuvant therapy, and further studies are needed to define TD and demonstrate its pathological mechanism for the apparent downstaging caused by receiving neoadjuvant therapy. Thirdly, the data for local cohort is inadequate to validate the conclusion obtained from SEER cohort. Furthermore, real-world multicenter studies are needed to further validate our finding.

## 5.Conclusion

Our findings revealed patients with TDs exhibited more aggressive tumors, characterized by advanced T staging (T3&T4), N staging (N2), perineural invasion, and more advanced TNM stage. TDs was identified as a poor prognostic factor in stage III CRC patients in this study, with the co-existence of TDs and lymph node (LN) metastasis associated the worst prognosis. Additionally, patients with a single LN positive had a significantly better overall survival (OS) than those with a single TD positive. Furthermore, a pairwise comparison showed no statistically significant difference between TD+N1a/b and N1c groups, while the OS of TD-LN+ (TD- N1a/b) patients was the most favorable within the N1 stage. Collectively, the aforementioned findings highlight the value of Tumor deposits is significantly underestimated in the AJCC TNM staging system and indicate the urgent requirement for an improved staging system to guide the treatment strategy of stage III colorectal cancer patients.

## Supporting information

S1 Fig Overall survival by chemotherapy in stage III subgroups. Among the CRC patients with stage II (A), IIA (B), IB (C) and IIIC (D) in S EER cohort, the Kaplan-Meier curves were performed to describe the significant difference stratified by chemotherapy in overall survival, and log-rank tests were used to compare the cumulative survival distributions.
(TIF)

S1 Table The comparison of clinicodemographic data for CRC patients with stage III in local cohort. (DOCX)

S2 Table Overall univariate and multivariate analysis for CRC patients with stage III in local cohort. (DOCX)

S1 Data (CSV)

S2 Data (XLSX)

## References

[1] Wong-Chong N, Motl J, Hwang G, Nassif GJ Jr, Albert MR, Monson JRT, et al. Impact of Tumor Deposits on Oncologic Outcomes in Stage III Colon Cancer. Dis Colon Rectum. 2018;61(9):1043–52. doi: 10.1097/DCR.0000000000001152 30086053

[2] Siegel RL, Wagle NS, Cercek A, Smith RA, Jemal A. Colorectal cancer statistics, 2023. CA Cancer J Clin. 2023;73(3):233–54. doi: 10.3322/caac.21772 36856579

[3] Li N, Lu B, Luo C, Cai J, Lu M, Zhang Y, et al. Incidence, mortality, survival, risk factor and screening of colorectal cancer: A comparison among China, Europe, and northern America. Cancer Lett. 2021;522:255–68. doi: 10.1016/j.canlet.2021.09.034 34563640

[4] Zheng R, Zhang S, Zeng H, Wang S, Sun K, Chen R, et al. Cancer incidence and mortality in China, 2016. Journal of the National Cancer Center. 2022;2(1):1–9. doi: 10.1016/j.jncc.2022.02.002 39035212 PMC11256658

[5] Kastrinos F, Kupfer SS, Gupta S. Colorectal Cancer Risk Assessment and Precision Approaches to Screening: Brave New World or Worlds Apart? Gastroenterology. 2023;164(5):812–27. doi: 10.1053/j.gastro.2023.02.021 36841490 PMC10370261

[6] Song M, Garrett WS, Chan AT. Nutrients, foods, and colorectal cancer prevention. Gastroenterology. 2015;148(6):1244–60.e16. doi: 10.1053/j.gastro.2014.12.035 25575572 PMC4409470

[7] Lawler T, Walts ZL, Steinwandel M, Lipworth L, Murff HJ, Zheng W, et al. Type 2 Diabetes and Colorectal Cancer Risk. JAMA Netw Open. 2023;6(11):e2343333. doi: 10.1001/jamanetworkopen.2023.43333 37962884 PMC10646729

[8] Jiang Y, Ben Q, Shen H, Lu W, Zhang Y, Zhu J. Diabetes mellitus and incidence and mortality of colorectal cancer: a systematic review and meta-analysis of cohort studies. Eur J Epidemiol. 2011;26(11):863–76. doi: 10.1007/s10654-011-9617-y 21938478

[9] Karki R, Man SM, Kanneganti TD. Inflammasomes and Cancer. Cancer Immunol Res. 2017;5(2):94–9. doi: 10.1158/2326-6066.CIR-16-0269 28093447 PMC5593081

[10] Sun Y, Hu H, Liu Z, Xu J, Gao Y, Zhan X, et al. Macrophage STING signaling promotes NK cell to suppress colorectal cancer liver metastasis via 4-1BBL/4-1BB co-stimulation. J Immunother Cancer. 2023;11(3). doi: 10.1136/jitc-2022-006481 36927529 PMC10030919

[11] Wang X, Cheng W, Dou X, Tan F, Yan S, Zhou Z, et al. The new ’coN’ staging system combining lymph node metastasis and tumour deposit provides a more accurate prognosis for TNM stage III colon cancer. Cancer Med. 2023;12(3):2538–50. doi: 10.1002/cam4.5099 35912894 PMC9939212

[12] Pyo DH, Kim SH, Ha SY, Yun SH, Cho YB, Huh JW, et al. Revised Nodal Staging Integrating Tumor Deposit Counts With Positive Lymph Nodes in Patients With Stage III Colon Cancer. Ann Surg. 2023;277(4):e825–e31. doi: 10.1097/SLA.0000000000005355 34954753

[13] Nagtegaal ID, Tot T, Jayne DG, McShane P, Nihlberg A, Marshall HC, et al. Lymph nodes, tumor deposits, and TNM: are we getting better? J Clin Oncol. 2011;29(18):2487–92. doi: 10.1200/JCO.2011.34.6429 21555695

[14] Nagtegaal ID, Knijn N, Hugen N, Marshall HC, Sugihara K, Tot T, et al. Tumor Deposits in Colorectal Cancer: Improving the Value of Modern Staging-A Systematic Review and Meta-Analysis. J Clin Oncol. 2017;35(10):1119–27. doi: 10.1200/JCO.2016.68.9091 28029327

[15] Cohen R, Shi Q, Meyers J, Jin Z, Svrcek M, Fuchs C, et al. Combining tumor deposits with the number of lymph node metastases to improve the prognostic accuracy in stage III colon cancer: a post hoc analysis of the CALGB/SWOG 80702 phase III study (Alliance)(☆). Ann Oncol. 2021;32(10):1267–75. doi: 10.1016/j.annonc.2021.07.009 34293461 PMC8719434

[16] Rock JB, Washington MK, Adsay NV, Greenson JK, Montgomery EA, Robert ME, et al. Debating deposits: an interobserver variability study of lymph nodes and pericolonic tumor deposits in colonic adenocarcinoma. Arch Pathol Lab Med. 2014;138(5):636–42. doi: 10.5858/arpa.2013-0166-OA 23902577 PMC3935980

[17] Wang Y, Zhang J, Zhou M, Yang L, Wan J, Shen L, et al. Poor prognostic and staging value of tumor deposit in locally advanced rectal cancer with neoadjuvant chemoradiotherapy. Cancer Med. 2019;8(4):1508–20. doi: 10.1002/cam4.2034 30790459 PMC6488131

[18] Song YX, Gao P, Wang ZN, Liang JW, Sun Z, Wang MX, et al. Can the tumor deposits be counted as metastatic lymph nodes in the UICC TNM staging system for colorectal cancer? PLoS One. 2012;7(3):e34087. doi: 10.1371/journal.pone.0034087 22461900 PMC3312887

[19] Li J, Yang S, Hu J, Liu H, Du F, Yin J, et al. Tumor deposits counted as positive lymph nodes in TNM staging for advanced colorectal cancer: a retrospective multicenter study. Oncotarget. 2016;7(14):18269–79. doi: 10.18632/oncotarget.7756 26934317 PMC4951287

[20] Yang J, Xing S, Li J, Yang S, Hu J, Liu H, et al. Novel lymph node ratio predicts prognosis of colorectal cancer patients after radical surgery when tumor deposits are counted as positive lymph nodes: a retrospective multicenter study. Oncotarget. 2016;7(45):73865–75. doi: 10.18632/oncotarget.12076 27655716 PMC5342019

[21] Jin M, Roth R, Rock JB, Washington MK, Lehman A, Frankel WL. The impact of tumor deposits on colonic adenocarcinoma AJCC TNM staging and outcome. Am J Surg Pathol. 2015;39(1):109–15. doi: 10.1097/PAS.0000000000000320 25229767 PMC4267920

[22] Hankey BF, Ries LA, Edwards BK. The surveillance, epidemiology, and end results program: a national resource. Cancer Epidemiol Biomarkers Prev. 1999;8(12):1117–21. 10613347

[23] Agger E, Jörgren F, Jöud A, Lydrup ML, Buchwald P. Negative Prognostic Impact of Tumor Deposits in Rectal Cancer: A National Study Cohort. Ann Surg. 2023;278(3):e526–e33. doi: 10.1097/SLA.0000000000005755 36538637

[24] Wu WX, Zhang DK, Chen SX, Hou ZY, Sun BL, Yao L, et al. Prognostic impact of tumor deposits on overall survival in colorectal cancer: Based on Surveillance, Epidemiology, and End Results database. World J Gastrointest Oncol. 2022;14(9):1699–710. doi: 10.4251/wjgo.v14.i9.1699 36187391 PMC9516655

[25] Atherton GJ, McCaul JA, Williams SA. Medical emergencies in general dental practice in Great Britain. Part 1: Their prevalence over a 10-year period. Br Dent J. 1999;186(2):72–9. doi: 10.1038/sj.bdj.4800023 10079576

[26] Lin Q, Wei Y, Ren L, Zhong Y, Qin C, Zheng P, et al. Tumor deposit is a poor prognostic indicator in patients who underwent simultaneous resection for synchronous colorectal liver metastases. Onco Targets Ther. 2015;8:233–40. doi: 10.2147/OTT.S71414 25653544 PMC4309783

[27] Goldstein NS, Turner JR. Pericolonic tumor deposits in patients with T3N+MO colon adenocarcinomas: markers of reduced disease free survival and intra-abdominal metastases and their implications for TNM classification. Cancer. 2000;88(10):2228–38. 10820343

[28] Arrichiello G, Pirozzi M, Facchini BA, Facchini S, Paragliola F, Nacca V, et al. Beyond N staging in colorectal cancer: Current approaches and future perspectives. Front Oncol. 2022;12:937114. doi: 10.3389/fonc.2022.937114 35928863 PMC9344134

[29] Khan H, Radomski SN, Siddiqi A, Zhou N, Paneitz DC, Johnston FM, et al. Tumor deposits are associated with a higher risk of peritoneal disease in non-metastatic colorectal cancer patients. J Surg Oncol. 2023;127(6):975–82. doi: 10.1002/jso.27207 36790093 PMC10079576

[30] Pricolo VE, Steingrimsson J, McDuffie TJ, McHale JM, McMillen B, Shparber M. Tumor Deposits in Stage III Colon Cancer: Correlation With Other Histopathologic Variables, Prognostic Value, and Risk Stratification-Time to Consider "N2c". Am J Clin Oncol. 2020;43(2):133–8. doi: 10.1097/COC.0000000000000645 31764018 PMC7004443

[31] Delattre JF, Cohen R, Henriques J, Falcoz A, Emile JF, Fratte S, et al. Prognostic Value of Tumor Deposits for Disease-Free Survival in Patients With Stage III Colon Cancer: A Post Hoc Analysis of the IDEA France Phase III Trial (PRODIGE-GERCOR). J Clin Oncol. 2020;38(15):1702–10. doi: 10.1200/JCO.19.01960 32167864

[32] Mirkin KA, Kulaylat AS, Hollenbeak CS, Messaris E. Prognostic Significance of Tumor Deposits in Stage III Colon Cancer. Ann Surg Oncol. 2018;25(11):3179–84. doi: 10.1245/s10434-018-6661-9 30083832

[33] Wünsch K, Müller J, Jähnig H, Herrmann RA, Arnholdt HM, Märkl B. Shape is not associated with the origin of pericolonic tumor deposits. Am J Clin Pathol. 2010;133(3):388–94. doi: 10.1309/AJCPAWOLX7ADZQ2K 20154277

[34] Lino-Silva LS, Xinaxtle DL, Salcedo-Hernández RA. Tumor deposits in colorectal cancer: the need for a new "pN" category. Ann Transl Med. 2020;8(12):733. doi: 10.21037/atm.2020.03.175 32647658 PMC7333091

[35] Zheng X, Cen W, Zhu J, Ye L. Prognostic Value of Tumor Deposits in Stage III Colorectal Cancer Patients with Different N Stages: A Population-Based, Retrospective, Cohort Study. Ann Surg Oncol. 2023;30(13):8067–73. doi: 10.1245/s10434-023-14338-x 37782414

[36] Brouwer NPM, Nagtegaal ID. Tumor deposits improve staging in colon cancer: what are the next steps? Ann Oncol. 2021;32(10):1209–11. doi: 10.1016/j.annonc.2021.08.1751 34416364

[37] Cao H, Xu E, Liu H, Wan L, Lai M. Epithelial-mesenchymal transition in colorectal cancer metastasis: A system review. Pathol Res Pract. 2015;211(8):557–69. doi: 10.1016/j.prp.2015.05.010 26092594

[38] De Smedt L, Palmans S, Andel D, Govaere O, Boeckx B, Smeets D, et al. Expression profiling of budding cells in colorectal cancer reveals an EMT-like phenotype and molecular subtype switching. Br J Cancer. 2017;116(1):58–65. doi: 10.1038/bjc.2016.382 27884016 PMC5220148

[39] Quagliariello V, D’Aiuto G, Iaffaioli RV, Berretta M, Buccolo S, Iovine M, et al. Reasons why COVID-19 survivors should follow dietary World Cancer Research Fund/American Institute for Cancer Research (WCRF/AICR) recommendations: from hyper-inflammation to cardiac dysfunctions. Eur Rev Med Pharmacol Sci. 2021;25(10):3898–907. doi: 10.26355/eurrev_202105_25957 34109598

[40] Hirota SA, Ng J, Lueng A, Khajah M, Parhar K, Li Y, et al. NLRP3 inflammasome plays a key role in the regulation of intestinal homeostasis. Inflamm Bowel Dis. 2011;17(6):1359–72. doi: 10.1002/ibd.21478 20872834 PMC3026862

[41] Allen IC, TeKippe EM, Woodford RM, Uronis JM, Holl EK, Rogers AB, et al. The NLRP3 inflammasome functions as a negative regulator of tumorigenesis during colitis-associated cancer. J Exp Med. 2010;207(5):1045–56. doi: 10.1084/jem.20100050 20385749 PMC2867287

