# Supplementary figures and images for: Negative prognostic impact of tumor deposits in stage III colorectal cancer patients
Source: PLoS One. 2024 Sep 26;19(9):e0310327. doi: 10.1371/journal.pone.0310327 (PMC11426431; doi:10.1371/journal.pone.0310327)

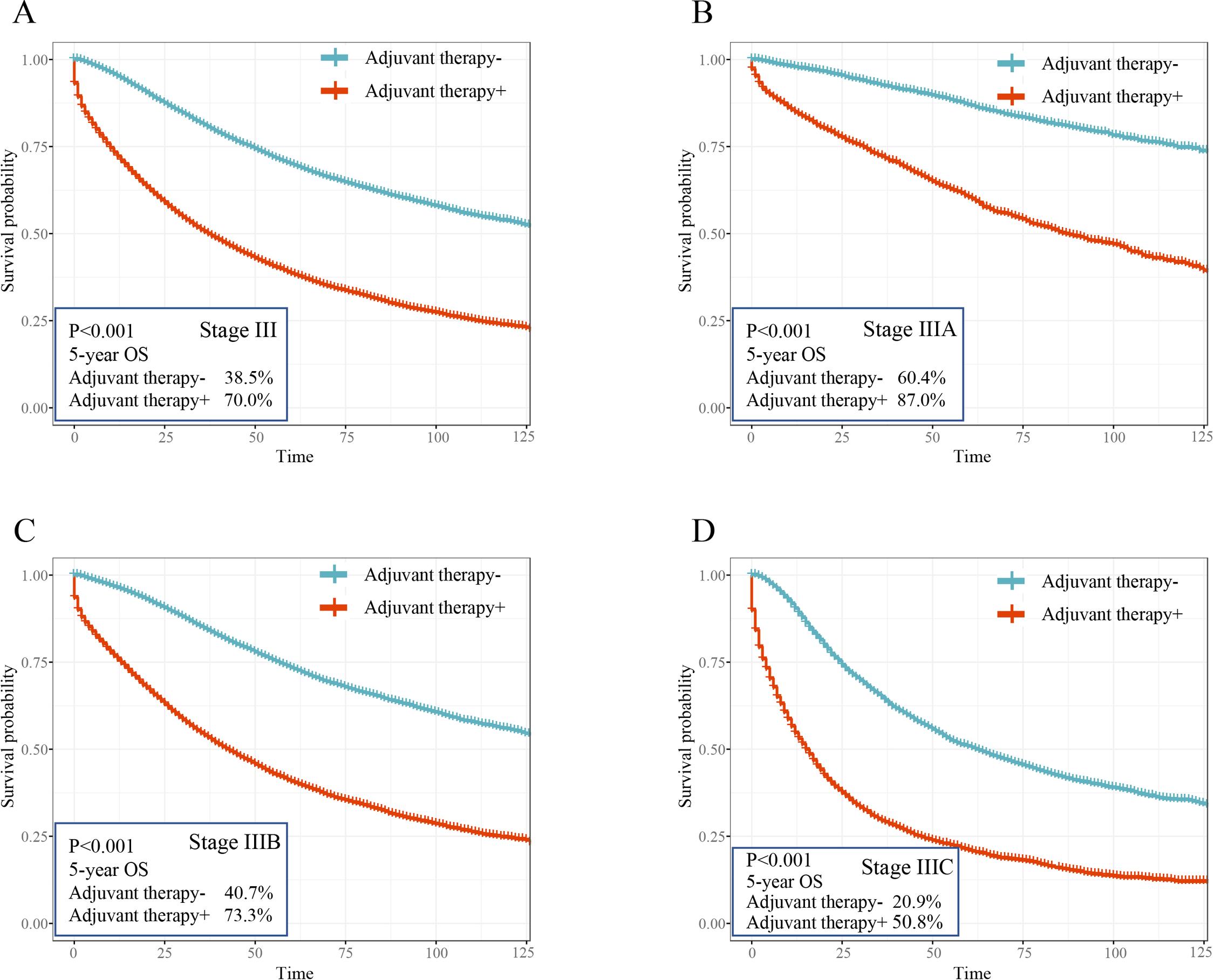

Supplement: S1 Fig — Among the CRC patients with stage II (A), IIA (B), IB (C) and IIIC (D) in S EER cohort, the Kaplan-Meier curves were performed to describe the significant difference stratified by chemotherapy in overall survival, and log-rank tests were used to compare the cumulative survival distributions. (TIF) [file pone.0310327.s001.tif]
