# Supplementary material for: Negative prognostic impact of tumor deposits in stage III colorectal cancer patients
Source: PLoS One. 2024 Sep 26;19(9):e0310327. doi: 10.1371/journal.pone.0310327 (PMC11426431; doi:10.1371/journal.pone.0310327)
Supplement: S1 Table — (DOCX) [file pone.0310327.s002.docx]

**S1 Table. The comparison of clinicodemographic data for CRC patients with stage III in local cohort.**

| **Characteristics** | **Overall N=3887(%)** | **Tumor deposits and Lymph nodes metastasis** | | | ***P*-value** |
| --- | --- | --- | --- | --- | --- |
|  |  | **TD-LN+ N=2627(%)** | **TD+LN- N=285(%)** | **TD+LN+ N=975(%)** |  |
| **Gender** |  |  |  |  | 0.079 |
| Female | 1565(40.3) | 1066(40.6) | 97(34.0) | 402(41.2) |  |
| male | 2322(59.7) | 1561(59.4) | 188(66.0) | 573(58.8) |  |
| **Age（years）** |  |  |  |  | 0.516 |
| ＜65 | 2330(59.9) | 1591(60.6) | 166(58.2) | 573(58.8) |  |
| ≥65 | 1557(40.1) | 1036(39.4) | 119(41.8) | 402(41.2) |  |
| **Schistosomiasis**  Yes  No  **Hepatitis**  Yes  No  **Diabetes**  Yes  No  **Hypertension**  Yes  No  **Site** | 3882(99.9)  5(0.0)  3827(98.5)  60(1.5)  3600(92.6)  287(7.4)  3121(80.3)  766(19.7) | 2622(99.8)  5(0.2)  2582(98.3)  45(1.7)  2434(92.7)  193(7.3)  2113(80.4)  514(19.6) | 285(100.0)  0(0.0)  280(98.2)  5(1.8)  260(91.2)  25(8.8)  231(81.1)  54(18.9) | 975(100.0)  0(0.0)  965(99.0)  10(1.0)  906(92.9)  69(7.1)  777(79.7)  198(20.3) | 0.301  0.317  0.624  0.836  <0.001 |
| Left colon | 588(15.1) | 403(15.2) | 40(14.0) | 145(14.9) |  |
| Right colon | 862(22.2) | 641(24.4) | 38(13.3) | 183(18.8) |  |
| Rectum | 2437(62.7) | 1583(60.3) | 207(72.6) | 647(66.4) |  |
| **T stage** |  |  |  |  | <0.001 |
| T1&T2 | 396(10.2) | 343(13.1) | 15(5.3) | 38(3.9) |  |
| T3&T4 | 3491(89.8) | 2284(86.9) | 270(94.7) | 937(96.1) |  |
| **N stage** |  |  |  |  | <0.001 |
| N1 | 2581(66.4) | 1782(67.8) | 285(100.0) | 514(52.7) |  |
| N2 | 1306(33.6) | 845(32.2) | 0(0) | 461(47.3) |  |
| **TNM stage** |  |  |  |  | <0.001 |
| IIIA | 337(8.7) | 297(11.3) | 15(5.3) | 25(2.6) |  |
| IIIB | 2346(60.4) | 1582(60.2) | 258(90.5) | 506(51.9) |  |
| IIIC | 1204(31.0) | 748(28.5) | 12(4.2) | 444(45.5) |  |
| **Perineural invasion** |  |  |  |  | <0.001 |
| Positive | 1535(39.5) | 921(35.1) | 95(33.3) | 519(53.2) |  |
| Negative | 2352(60.5) | 1706(64.9) | 190(66.7) | 456(46.8) |  |
| **Preoperative CEA** |  |  |  |  | <0.001 |
| Positive | 1427(36.7) | 864(32.9) | 114(40.0) | 449(46.1) |  |
| Negative | 2137(55.0) | 1531(58.3) | 158(55.4) | 448(45.9) |  |
| Unknown | 323(8.3) | 232(8.8) | 13(4.6) | 78(8.0) |  |
| **Adjuvant chemoradiotherapy** |  |  |  |  | 0.862 |
| Yes | 2957(76.1) | 1993(75.9) | 216(75.8) | 748(76.7) |  |
| No | 930(23.9) | 634(24.1) | 69(24.2) | 227(23.3) |  |
| **LN Dissection** |  |  |  |  | <0.001 |
| ≤11 | 371(9.5) | 194(7.4) | 50(17.5) | 127(13.0) |  |
| ≥12 | 3480(89.5) | 2402(91.4) | 235(82.5) | 843(86.5) |  |
| Unknown | 36(0.9) | 31(1.2) | 0(0) | 5(0.5) |  |
