# Supplementary material for: Negative prognostic impact of tumor deposits in stage III colorectal cancer patients
Source: PLoS One. 2024 Sep 26;19(9):e0310327. doi: 10.1371/journal.pone.0310327 (PMC11426431; doi:10.1371/journal.pone.0310327)
Supplement: S2 Table — (DOCX) [file pone.0310327.s003.docx]

**S2 Table. Overall univariate and multivariate analysis for CRC patients with stage III in local cohort.**

| **Characteristics** | **Univariate COX regression** | | | **Multivariate COX regression** | | |
| --- | --- | --- | --- | --- | --- | --- |
|  | **HR** | **CI** | ***P* value** | **HR** | **CI** | ***P* value** |
| **Gender** |  |  |  |  |  |  |
| Female | 1 |  |  |  |  |  |
| male | 0.969 | 0.846-1.110 | 0.654 |  |  |  |
| **Age（years）** |  |  |  |  |  |  |
| ＜65 | 1 |  |  | 1 |  |  |
| ≥65 | 1.665 | 1.456-1.905 | <0.001 | 1.480 | 1.227-1.716 | <0.001 |
| **Schistosomiasis**  Yes  No  **Hepatitis**  Yes  No  **Diabetes**  Yes  No  **Hypertension**  Yes  No  **T stage** | 1  0.05  1  0.793  1  1.130  1  1.144 | 0.000-14994.816  0.437-1.438  0.879-1.453  0.964-1.357 | 0.641  0.445  0.340  0.129 |  |  |  |
| T1&T2 | 1 |  |  | 1 |  |  |
| T3&T4 | 2.799 | 2.030-3.860 | <0.001 | 1.648 | 1.182-2.298 | 0.003 |
| **N stage** |  |  |  |  |  |  |
| N1 | 1 |  |  | 1 |  |  |
| N2 | 2.231 | 1.952-2.551 | <0.001 | 1.791 | 1.553-2.066 | <0.001 |
| **Tumor Deposits** |  |  |  |  |  |  |
| No | 1 |  |  | 1 |  |  |
| Yes | 2.231 | 1.952-2.551 | <0.001 | 1.911 | 1.656-2.204 | <0.001 |
| **Perineural invasion** |  |  |  |  |  |  |
| Negative | 1 |  |  | 1 |  |  |
| Positive | 1.946 | 1.702-2.225 | <0.001 | 1.740 | 1.506-2.011 | <0.001 |
| **Preoperative CEA** |  |  |  |  |  |  |
| Negative | 1 |  |  | 1 |  |  |
| Positive | 1.516 | 1.318-1.744 | <0.001 | 1.301 | 1.130-1.499 | <0.001 |
| **Adjuvant chemoradiotherapy** |  |  |  |  |  |  |
| No | 1 |  |  | 1 |  |  |
| Yes | 0.42 | 0.365-0.484 | <0.001 | 0.444 | 0.381-0.519 | <0.001 |
| **LN Dissection** |  |  |  |  |  |  |
| ≤11 | 1 |  |  |  |  |  |
| ≥12 | 0.836 | 0.682-1.024 | 0.083 |  |  |  |
|  |  |  |  |  |  |  |
|  |  |  |  |  |  |  |
